# Dermatofibrosarcoma Protuberans Presenting in a Patient With Neurofibromatosis Type 1: Potential Implications on Treatment

**DOI:** 10.7759/cureus.17675

**Published:** 2021-09-03

**Authors:** Bianca N Eubanks, Dawood A Tafti, Sabrina House, Nicholas Logemann

**Affiliations:** 1 Medicine, Uniformed Services University of the Health Sciences, Bethesda, USA; 2 Department of Radiology, San Antonio Military Medical Center, San Antonio, USA; 3 Department of Dermatology, McDonald Army Health Center, Newport News, USA; 4 Department of Dermatology, Walter Reed National Military Medical Center, Bethesda, USA

**Keywords:** dermatofibrosarcoma protuberans, neurofibromatosis type 1, imatinib mesylate, chromosomal translocation, chromosome 17

## Abstract

Dermatofibrosarcoma protuberans (DFSP) is a rare soft tissue sarcoma. Neurofibromatosis type 1 (NF1) is a neurocutaneous syndrome that affects multiple organ systems. We present the case of a 47-year-old African American male with a two-year history of a slowly enlarging right lower back lesion. Upon workup, the 3 × 2 cm mass was biopsied confirming a diagnosis of DFSP. This was identified in concert with axillary freckling, café-au-lait spots, and pedunculated plaques evaluated with biopsy. The findings were consistent with neurofibromas, leading to a new diagnosis of NF1. The patient was definitively treated with wide local excision of the DFSP lesion without tumor recurrence over six years. DFSP has a favorable prognosis when treated with wide local excision and negative surgical margins. However, lesions may recur with inadequate margins. Although deferred in our patient, treatment with imatinib mesylate, a tyrosine kinase inhibitor, may be employed in the setting of advanced disease, metastasis, positive surgical margins, or irresectable locations. Imatinib has also been used to treat NF1. Hence, we posit that the concomitant presentation of these two disease entities in our patient highlights a potentially unique treatment with imatinib mesylate. To our knowledge, this is the second reported case of both entities in the same patient.

## Introduction

Dermatofibrosarcoma protuberans (DFSP) is an uncommon, slow-growing, intermediate-grade tumor in the dermis and subcutaneous fat [1]. It most commonly presents as a firm, protuberant growth in adults [1]. DFSP is also characterized by its locally aggressive behavior with a low propensity for metastasis [1]. Although metastasis is rare, it most commonly occurs in the lungs [1]. DFSP is a soft tissue tumor that occurs in 0.8 to 4.5 cases per million persons per year, making its clinical manifestation with neurofibromatosis type 1 (NF1), an autosomal dominant disorder defined by the presence of at least two of seven criteria such as neurofibromas and iris pigmented hamartomas, a unique presentation [1,2]. There are two main types of neurofibromas, namely, cutaneous and plexiform, that differ in multiple respects, including their cumulative growth and malignant potential [3].

Although the pathogenic mechanism of DFSP is not fully understood, the pathogenesis is thought to involve a chromosomal fusion secondary to translocation between chromosomes 17 and 22. Over 90% of patients diagnosed with DFSP harbor the t(17;22)(q22;q13) translocation that causes an upregulation of platelet-derived growth factor (PDGF) and subsequent tumor formation [1]. This translocation represents the fusion of *collagen type 1 alpha 1 *gene (*COL1A1*)on chromosome 17 and *platelet-derived growth factor-beta polypeptide *gene(*PDGFB*) that ultimately results in an overproduction of PDGF [1]. Of note, the *NF1 *gene, a tumor-suppressor gene that codes for neurofibromin type 1, is also located on chromosome 17 [2]. Although the gold standard for the treatment of DFSP is surgical removal with clear margins, the use of imatinib mesylate, an oral tyrosine kinase inhibitor, has shown promise in the treatment of locally advanced, recurrent, or metastatic disease [1,4]. Given observation in mouse studies involving NF1 tumors, with subsequent evaluation in clinical trials, imatinib mesylate has been hypothesized as a treatment option in patients with a high tumor burden, especially in plexiform neurofibromas that are not amenable to resection or responsive to chemoradiotherapy [5]. We theorize that in the setting of our patient who is comorbid with both DFSP and NF1, imatinib mesylate could be a potential treatment as it has been used with some success in both diseases [2,5].

## Case presentation

A 47-year-old African American male with no significant medical history presented to his primary care provider for concerns of a two-year history of a slowly enlarging right lower back lesion with infrequent lower back spasms that were well-controlled with cyclobenzaprine and ibuprofen. The patient denied any systemic symptoms or other new or worsening dermatologic conditions. Physical examination was significant for a painless 3 × 2 cm mass located on the right lower back.

**Figure 1:**
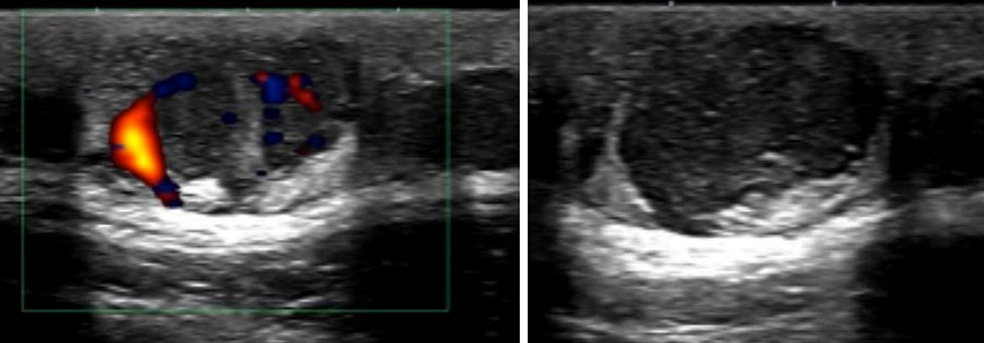
Sonographic image of the dermatofibrosarcoma protuberans lesion showing heterogeneous echogenicity and vascularity of the mass, as evidenced by Doppler flow.

**Figure 2:**
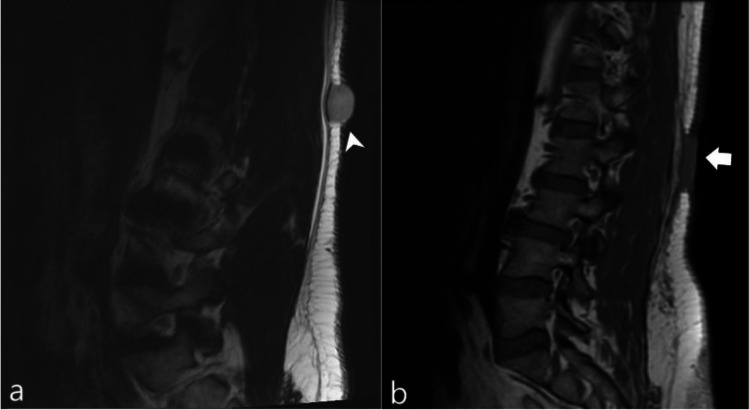
(a) Sagittal T2-weighted MRI illustrates the dermatofibrosarcoma protuberans lesion (arrowhead) as a well-circumscribed mass at the level of the L1-L2 disk space confined to the subcutaneous tissue. The mass measures 2.2 cm in the craniocaudal dimension by 1.6 cm in the anteroposterior dimensions. There is no evidence of underlying soft tissue invasion. (b) Sagittal T1 sequence demonstrates post-surgical T1 intermediate signal changes of the subcutaneous soft tissues of the back at the dermatofibrosarcoma protuberans surgical bed (arrow) approximately six months post-excision. MRI: magnetic resonance imaging

**Figure 3:**
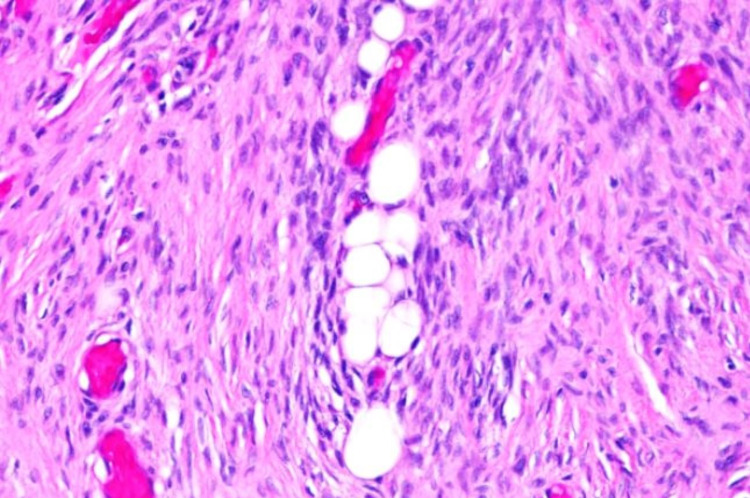
Hematoxylin and eosin stain (200×) histopathological image of the dermatofibrosarcoma protuberans lesion showing a storiform pattern of bland spindle cells with low mitotic activity that are infiltrating into the subcutaneous fat.

Sonographic examination revealed a heterogenous and vascularized tumor that was originally thought to be a simple lipoma based on history and physical examination findings (Figure 1). A magnetic resonance imaging (MRI) study of the lesion demonstrated a well-circumscribed mass localized to the subcutaneous tissue without note in the radiology report of distant metastasis or deeper fascial involvement (Figure 2, Panel a). A subsequent ultrasound-guided biopsy demonstrated histopathologic findings of spindle cells in a storiform pattern with low mitotic activity within the subcutaneous fat, consistent with the diagnosis of DFSP (Figure 3). Based on this diagnosis, the patient was referred to a tertiary care center for definitive treatment to include wide local excision; treatment with imatinib mesylate was deferred. A follow-up MRI at six months post-surgery demonstrated no evidence of recurrence at the surgical bed (Figure 2, Panel b).

With the diagnosis of DFSP, the patient was referred to dermatology. At the dermatology clinic, the patient was noted to have at least six hyperpigmented patches consistent with café-au-lait spots scattered over the left frontal scalp, left chest, left flank, left lateral thigh, and left upper back. The physical examination further revealed left axillary freckling and multiple soft 1-2 cm pedunculated plaques consistent with neurofibromas located on the bilateral shoulders and left upper back. Biopsy of one of the lesions confirmed a neurofibroma without plexiform features. These findings confirmed a new diagnosis of NF1. The patient had no other systemic involvement and is now six years from DFSP resection and remains tumor-free.

## Discussion

**Figure 4:**
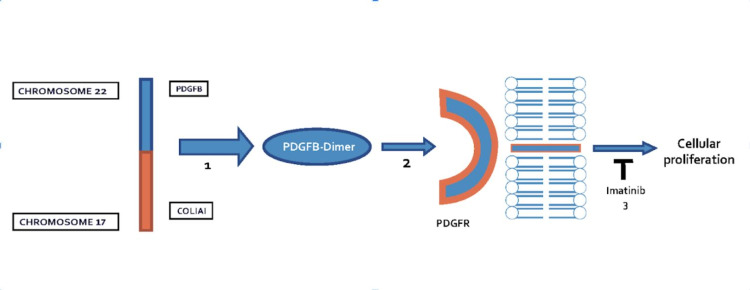
Fusion of the COL1A1 gene on chromosome 17 and the PDGFB gene on chromosome 22 leads to the production of PDGFB-dimer. This leads to the activation of PDGFR triggering a signal cascade for cellular proliferation. Imatinib acts as a tyrosine kinase inhibitor blocking this signal transduction intracellularly. *COL1A1*: *collagen type 1 alpha 1*; *PDGFB*: *platelet-derived growth factor-beta polypeptide*; PDGFR: platelet-derived growth factor receptor

The traditional gold standard for the treatment of a DFSP lesion is wide local excision with clear margins. However, some recent studies suggest Mohs micrographic surgery is a better therapeutic option compared to wide local excision, with lower rates of local recurrence given the ability of DFSP lesions to be more widespread than clinically apparent [1]. However, if a case is inoperable, imatinib mesylate can offer an alternative treatment option [1,6]. Imatinib mesylate works by inhibiting the PDGF receptor, a cell surface tyrosine kinase receptor (Figure 4). This leads to a decrease in tumor progression and eventually apoptosis, with a response rate to treatment reported as high as 65% [1]. Adverse side effects, such as gastrointestinal upset and fatigue, have been noted and should be taken into consideration if planning to administer this medication [1,6].

Patients with plexiform neurofibromas in NF1 have also been successfully treated with imatinib mesylate [3,5]. The *NF1 *gene is a tumor-suppressor gene that codes for neurofibromin, and mutation may manifest in cutaneous tumors such as plexiform neurofibromas [3]. Tumor formation in cutaneous and plexiform neurofibromas of NF1 requires a homozygous, second-hit mutation in Schwann cells; however, this event is not enough for tumorigenesis [3]. Mouse studies have implicated that a heterozygous mutation in mast cells is also necessary for NF1 tumor formation in conjunction with the homozygous mutation in Schwann cells, as these mutated Schwann cells secrete chemotactic kit ligand (c-kit) [3]. Heightened levels of c-kit result in the increased number and persistence of mast cells [3]. Other cell types such as fibroblasts, adipocytes, perineural cells, and epithelial cells have also been indicated as part of a potential etiology, although they are components of normal cellular processes [3]. Unlike cutaneous neurofibromas which undergo a later second-hit mutation and therefore commonly manifest during adulthood, plexiform neurofibroma inactivation is thought to occur earlier in embryonic cells, accounting for its presentation during childhood [3].

**Figure 5:**
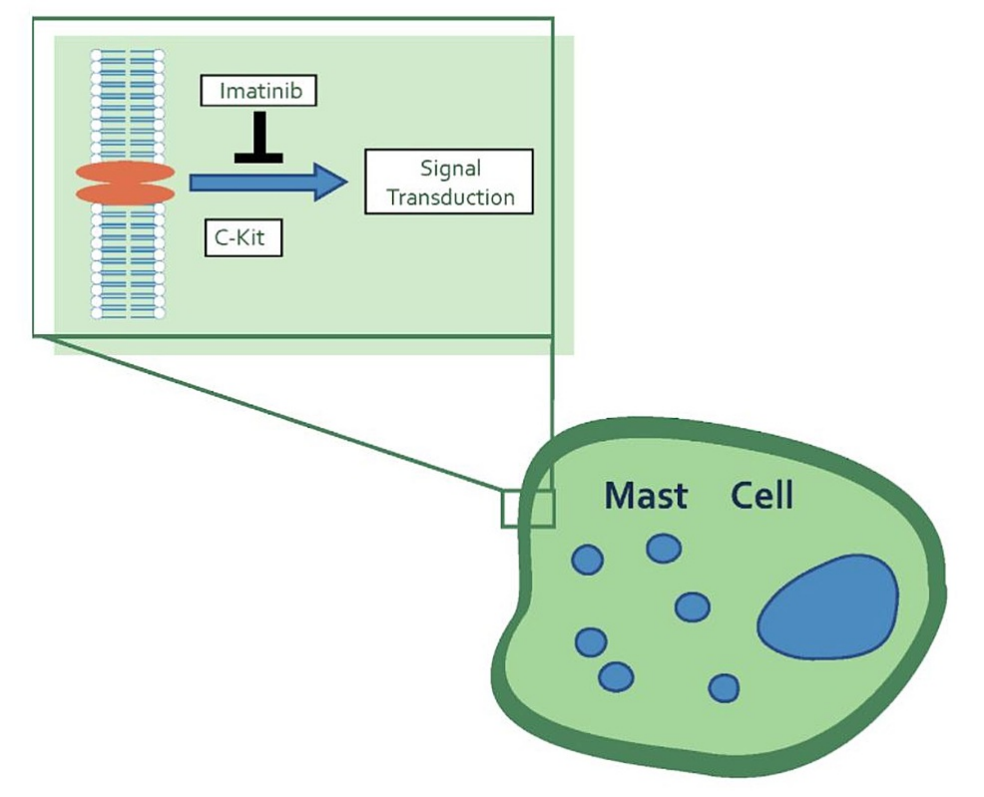
Imatinib blocks signal transduction at c-kit in mast cells. Mast cells play a role in the pathogenesis of NF1 due to their role in the tumor microenvironment necessary for tumorigenesis in neurofibromas. c-kit: chemotactic kit ligand; NF1: neurofibromatosis type 1

Similar to DFSP, plexiform neurofibromas are slow-growing tumors that do not respond well to chemotherapy [5]. Imatinib mesylate is reported as a potential inhibitor of c-kit in addition to PDGFB and may explain NF1 treatment response (Figure 5) [3]. The diagnosis of both DFSP and NF1 in this patient highlights a potentially novel approach to the treatment of both diseases concurrently with imatinib mesylate [5]. However, no research supporting a common underlying pathoetiology of the comorbidities has yet been established. We wonder if DFSP may be underdiagnosed in patients presenting concurrently with NF1 as patients may have many lesions which are assumed to be neurofibromas and never biopsied.

## Conclusions

This case report illustrates a rare case of DFSP occurring in the context of a patient with NF1. While these two disease entities involve aberrations in chromosome 17 and respond in some capacity to treatment with imatinib mesylate as previously discussed, their pathogenesis is currently unrelated. Only one other case of DFSP in a patient with NF1 has been described in a four-year-old patient with a DFSP involving the left temple (Poster: D. Viskochil, L. Randall. Dermatofibrosarcoma protuberans in neurofibromatosis type 1 (NF1). American Society of Human Genetics Annual Meeting 2011; 11-15 Oct 2011). Despite a lack of literature demonstrating related pathogenesis, imatinib mesylate has been utilized in both disease entities and could be worthy of further consideration as therapy in patients with DFSP in combination with NF1 in the setting of promising therapeutic responses to each individually.
